# Adaptation and Transcriptome Analysis of *Aureobasidium pullulans* in Corncob Hydrolysate for Increased Inhibitor Tolerance to Malic Acid Production

**DOI:** 10.1371/journal.pone.0121416

**Published:** 2015-03-20

**Authors:** Xiang Zou, Yongkang Wang, Guangwei Tu, Zhanquan Zan, Xiaoyan Wu

**Affiliations:** 1 College of Pharmaceutical Sciences, Chongqing Engineering Research Center for Pharmaceutical Process and Quality Control, Southwest University, Chongqing 400715, P.R China; 2 Key Laboratory of Biorheological Science and Technology (Chongqing University), Ministry of Education, Chongqing 400044, P.R China; Korea University, KOREA, REPUBLIC OF

## Abstract

Malic acid is a dicarboxylic acid widely used in the food industry, and is also a potential C4 platform chemical. Corncob is a low-cost renewable feedstock from agricultural industry. However, side-reaction products (furfural, 5-hydroxymethylfurfural (HMF), formic acid, and acetic acid) that severely hinder fermentation are formed during corncob pretreatment. The process for producing malic acid from a hydrolysate of corncob was investigated with a polymalic acid (PMA)-producing *Aureobasidium pullulans* strain. Under the optimal hydrolysate sugar concentration 110 g/L, *A*. *pullulans* was further adapted in an aerobic fibrous bed bioreactor (AFBB) by gradually increasing the sugar concentration of hydrolysate. After nine batches of fermentation, the production and productivity of malic acid reached 38.6 g/L and 0.4 g/L h, respectively, which was higher than that in the first batch (27.6 g/L and 0.29 g/L h, respectively). The adapted strain could grow under the stress of 0.5 g/L furfural, 3 g/L HMF, 2g/L acetic acid, and 0.5 g/L formic acid, whereas the wild type did not. Transcriptome analysis revealed that the differentially expressed genes were related to carbohydrate transport and metabolism, lipid transport and metabolism, signal transduction mechanism, redox metabolism, and energy production and conversion under 0.5 g/L furfural and 3 g/L HMF stress conditions. In total, 42 genes in the adapted strain were upregulated by 15-fold or more, and qRT-PCR also confirmed that the expression levels of key genes (i.e. *SIR*, *GSS*, *CYS*, and *GSR*) involved in sulfur assimilation pathway were upregulated by over 10-fold in adapted strain for cellular protection against oxidative stress.

## Introduction

Malic acid (2-hydroxybutanedioic acid) is a C4 dicarboxylic acid and an intermediate in the tricarboxylic acid (TCA) cycle. It is used predominantly in the food and beverage industries as an acidulant and taste enhancer/modifier, particularly in combination with artificial sweeteners [1]. Additionally, the U.S. Department of Energy has identified malic acid as a building block chemical that can be converted to various high-volume chemical products from renewable sources of carbohydrate and converted to various chemical products [2]. The future use of malic acid is predicted to be over 200,000 tons per year [3].

Compared with chemical synthesis or enzymatic hydration of fumarate to produce malic acid, considerable interest has been shown in the production of malic acid by microorganism fermentation. Numerous microorganisms—various *Aspergillus* species [4], *Zygosaccharomyces rouxii* [5], *Schizophyllum commune* [6], and engineered *Bacillus subtilis*, *Saccharomyces cerevisiae*, and *Escherichia coli* [7–10]—have been isolated or engineered to produce malic acid. However, microbial fermentation for direct malic acid production is usually limited by low product yield, titer, and productivity due to end-product inhibition. The by-products of other organic acids, such as succinic and acetic acid, also renders the downstream process of separating and purifying malic acid relatively complex and expensive [11]. In our previous work, we developed a novel process for malic acid production from polymalic acid (PMA) fermentation by *Aureobasidium pullulans* followed by acid hydrolysis. A high-yield PMA-producing strain was isolated and used to produce malic acid in a fed-batch fermentation, achieving high product (malic acid) titer of 142.2 g/L, productivity of 0.74 g/L h, and yield of 0.55 g/g glucose [12].

Malic acid production is relatively expensive when glucose is used as the carbon substrate. Currently, renewable biomass has been intensively investigated to produce bio-fuels and chemicals via the sugar platforms [13]. China is a major corn-producing country, and corncob by-products have a high content of hemicellulose from which fermentative sugar can be produced by acid or enzyme hydrolysis. Unfortunately, side-reaction products (furfural, 5-hydroxymethylfurfural (HMF), formic acid, and acetic acid) are formed during corncob pretreatment [14,15]. These undesirable inhibitory by-products are toxic to cells and can severely impair the fermentation process by inhibiting microbial growth and metabolism.

*A*. *pullulans* is a cosmopolitan yeast-like fungus showing substantial tolerance toward heavy metal ions, osmotic pressure, and extreme environment [16,17]. However, the cellular response of *A*. *pullulans* to inhibitor stress from hemicellulose hydrolysates is poorly understood. In this work, we optimized the production of malic acid from corncob hydrolysate, and enhanced the tolerance to inhibitors by culture adaptation in an aerobic fibrous bed bioreactor (AFBB). A highly adapted strain was successfully isolated from an AFBB. The underlying mechanism contributing to the improved malic acid production and the increased tolerance of the adapted strain were further elucidated by transcriptome analysis.

## Materials and Methods

### Cultures and media

The strain *A*. *pullulans* CCTCC M2012223 was isolated by our laboratory and can be obtained from the China Center for Type Culture Collection (Wuhan, China). Potato Dextrose Agar (PDA) slants were inoculated with cells and incubated at 25°C for 2 days, and then used for seed culture inoculation. For seed culture, the medium composition included (g/L): glucose 60, NH_4_NO_3_ 2, KH_2_PO_4_ 0.1, MgSO_4_ 0.1, ZnSO_4_ 0.1, KCl 0.5, CaCO_3_ 20 and corn steep liquor 1. The seed culture was grown in 500 mL shake flask containing 50 mL of liquid medium and incubated at 25°C on a rotary shaker (220 rpm) for 2 days. The fermentation medium composition included (g/L): hydrolysate of corn cob 50–130 (calculated by total sugar containing xylose and glucose), NH_4_NO_3_ 2, KH_2_PO_4_ 0.1, MgSO_4_ 0.1, ZnSO_4_ 0.1, KCl 0.5, and CaCO_3_ 30. The fermentation cultivation was inoculated with 10% (v/v) of the above seed culture medium and kept at 25°C and 220 rpm for 5 days.

### Preparation of corncob hydrolysate

Acid hydrolysis of corncob was carried out with 1.0% H_2_SO_4_ (V/V) at a solid (corncob) to liquid (acid) ratio of 1:10 (W/V), and the mixture was hydrolyzed by autoclaving at 121°C for 40 min. Then, cellulase (10000 U/g, from Aladdin, China) and xylanase (10000 U/g, from Aladdin, China) was added into the mixture at a loading of 0.01 g/g corncob, respectively, and hydrolyzed at 50°C for 48 h. After hydrolysis, the mixture was centrifuged at 2700×*g* for 15 min to remove the insolubles, and the supernatant was further concentrated in vacuum at 70°C for 1 h to prepare different sugar concentrations. The contents of main by-products in concentrated corncob hydrolysate are shown in Table 1. All trails were performed in triplicate.

**Table 1:** Compositions of different inhibitors in corncob hydrolysate with total sugar concentration 150 g/L.

Contents	Concentrations (g/L)
Glucose	96
Xylose	54
Furfural	0.14
HMF	2.34
Formic acid	0.1
Acetic acid	1.8

**Table 2:** Effects of initial sugar concentration of corncob hydrolysate on malic acid production in shake flasks.

Initial total sugar (g/L)	Residual sugar(g/L)	Cell biomass (g/L)	Malic acid*(g/L)	productivity(g/L h)	yield(g/g)
50	0	12.75±0.68	5.90±0.53	0.06±0.005	0.12
60	0	15.50±0.52	6.03±0.62	0.06±0.006	0.11
70	3.62	20.86±0.13	11.42±3.91	0.12±0.040	0.17
80	6.88	26.66±0.58	11.58±2.18	0.12±0.022	0.16
90	12.34	30.88±0.20	12.66±1.05	0.13±0.010	0.16
110	21.87	32.13±0.20	15.27±2.18	0.16±0.023	0.17
130	43.18	27.45±0.52	10.29±2.70	0.11±0.028	0.12

* Malic acid was obtained after hydrolysis of PMA with H_2_SO_4_.

### Optimization of initial sugar concentration from corncob hydrolysate in shake flask

To investigate the effect of initial sugar concentration on malic acid production in a shake flask, the total hydrolysate sugar levels of 50, 60, 70, 80, 90,110, 120, and 130 g/L were tested. The other components of fermentation medium were KH_2_PO_4_ 0.1, MgSO_4_·7H_2_O 0.1, KCl 0.5, ZnSO_4_ 0.1, and CaCO_3_ 30 g/L. The above seed culture (10%, v/v) was inoculated to 50 mL fermentation medium containing different initial residual sugar concentration. The initial pH value was set at 6.5, and the fermentation cultivation was then operated at 25°C and 220 rpm for 5 days. All trails were performed in triplicate.

### Effect of nitrogen sources on malic acid production

The effect of nitrogen sources on the malic acid production in a shake flask was studied by using 2 g/L of one of the following nitrogen source, i.e. peptone, yeast extract, NH_4_H_2_PO_4_, (NH_4_)_2_SO_4_, KNO_3_, and NH_4_NO_3_. The other culture medium components were hydrolysate of corn cob 110 (calculated by total sugar), KH_2_PO_4_ 0.1, MgSO_4_·7H_2_O 0.1, KCl 0.5, ZnSO_4_ 0.1, and CaCO_3_ 30 g/L. The initial pH value was set at 6.5, and all trails were performed in triplicate.

### Bioreactor setup and adaptation culture

The fermentation system consisted of a 0.5 L aerobic fibrous-bed bioreactor (AFBB) connected to 5 L fermenter connected with a recirculation loop and operated under well-mixed conditions for pH and temperature control. Same aeration rate as in free cell fermentation was used in the AFBB for oxygen supply. Details about the setup of the bioreactor have been given elsewhere [18]. The repeated-batch fermentations were carried out in the AFBB system to study the fermentation kinetics and gradually enhanced the concentration of hydrolysate sugar from 110 to 150 g/L to adapt the cells tolerance. Samples were taken every 12 h for the analyses of dry cell weight, the production of malic acid, and the residual sugar concentration. After 864 h continuous fermentation, the adapted cells in the AFBB were removed from the fibrous matrix by vortexing the matrix in sterile distilled water under aerobic conditions and isolated from a single colony on plate for further analysis.

### Cell tolerance to inhibitor stress

Adapted and original cells were cultured in serum tubes containing 10 mL of synthetic media with 20 g/L glucose and varying amounts of furfural, HMF, formic acid, and acetic acid. To evaluate the inhibitory effect on cell growth, the cell concentration was monitored by measuring the optical density at 600 nm with a spectrophotometer.

### Scanning electron microscopy

Adapted and original strains were subcultured in tubes for several generations. The cells were collected in the stationary phase. The samples were fixed in 2.5% (w/v) glutaraldehyde for 15 h at 4°C and rinsed with distilled water twice. The samples were processed through a progressive dehydration with 20–100% ethanol at 10% increment, dried with HMDS, and coated with gold/palladium. The samples were scanned and photographed with a Hitachi S-3400N scanning electron microscope at 15 kV.

### Total RNA purification

The total cellular RNA was purified from 2 mL the original and adapted strain culture, respectively. Briefly, the original and adapted strain cells from 2 mL culture broth were freezed in liquid nitrogen and re-suspended in the addition of 500 μL RB buffer supplemented with 2% (v/v) β-mercaptoethanol (β-ME). For complete lysis of *A*. *pullulans* cells, a 2 mL microcentrifuge tube was filled with 500 μL of water-saturated phenol and 100 μL sodium acetate, and then supplemented with 0.2 mL chloroform. Total RNA was purified from homogenized cells using an RNeasy mini kit (Qiagen, Germany) according to the manufacturer’s instructions. RNA quality was analyzed using a Nanochip 2100 bioanalyzer (Agilent Technologies Inc., USA), and RNA concentration was measured by NanoDrop 3300 (Thermo scientific, USA) according to the manufacturer’s instructions.

### Transcriptome sequencing and analysis

After the total RNA extraction and DNase I treatment (37°C and 30 min), magnetic beads with Oligo (dT) are used to isolate mRNA. Mixed with the fragmentation buffer, the mRNA is fragmented into short fragments. Then cDNA is synthesized using the mRNA fragments as templates. Short fragments are purified and resolved with EB buffer for end reparation and single nucleotide A (adenine) addition. After that, the short fragments are connected with adapters. The suitable fragments are selected for the PCR amplification as templates. During the QC steps, Agilent 2100 Bioanaylzer and ABI StepOnePlus Real-Time PCR System are used in quantification and qualification of the sample library. At last, the library was sequenced using Illumina HiSeq 2000 equipment.

In a comparison analysis, two-class unpaired method in the significant analysis of microarray software (SAM, version 3.02) was performed to identify significantly differentially expressed genes between furfural and HMF treated and control groups. Genes were determined to be significantly differentially expressed with a selection threshold of false discovery rate, FDR <5% and fold change ≥2. Raw date was log_2_-transformed and imported.

### Assay of cell biomass and residual total sugar

The cell density was determined by dry cell weight (DCW) method. Before the measurement, excess CaCO_3_ in the broth was eliminated with the addition of 1 M HCl. The cell suspension was centrifuged at 2700×*g* and then overnight drying at 105°C. The residual total sugar includes the xylose and glucose, and measured with a high performance liquid chromatograph (HPLC) equipped with an organic acid analysis column (Spursil C18-EP) and a refractive index detector (Shimadzu RID-10A) at 45°C. The eluent used was 5 mM H_2_SO_4_ at 0.6 mL/min [19].

### Assay of malic acid production

For analysis of malic acid, the fermentation broth was centrifuged and then 1 mL of resulted supernatant was mixed with 1 mL 2 M H_2_SO_4_ and incubated at 85°C for 8 h. After neutralization of the solution, the hydrolyzed sample was analyzed by HPLC (Hitachi L-2000, Japan) for its content of malic acid, using a Spursil C18-EP organic acid column eluted with 5 mM H_2_SO_4_ at 40°C and the flow rate of 0.6 mL/min [18].

### Quantitative RT-PCR

To confirm the gene transcription levels, four genes, sulfite reductase (SIR), glutathione synthase (GSS), cysteine synthase (CYS), and glutathione reductase (GSR), involved in sulfur assimilation pathway were tested by quantitative RT-PCR method. Besides the transcriptome analysis, total RNA from the adapted strain and original strain was also extracted using Trizol Reagent (Ambion, USA), respectively, and got cDNA using reverse transcriptase (Vazyme, USA). Primers of four genes were showed in S1 Table.The experiment was repeated three times, The PCR conditions were 3 min at 94°C, followed by 45 cycles of 30 s at 94°C, 20 s at 55°C, and 30s at 72°C. To check the specificity of the primers, a dissociation protocol was added after thermocycling, determining the dissociation of the PCR products from 60°C to 95°C (The dwell time was 15 s, and the temperature gradient was +0.5°C per cycle). The quantitative PCR assay was performed according to Sybr Green method (qPCR Master Mix, TaKaRa, Japan) using fluorescence quantitative PCR (Roche, USA).

## Results and Discussion

### Effect of the initial hydrolysate sugar concentration on malic acid production

Corncob is an abundant raw material with high (35%) hemicellulose content. After hydrolysis with dilute sulfuric acid, the corncobs released monomeric sugars, such as xylose, glucose, arabinose, etc. [20,21]. In addition to sugars, several microbial inhibitors, including furfural, HMF, acetic acid, and phenolic compounds were generated in the hydrolysis process. In the fermentation process, it is necessary to concentrate the hydrolysates in order to increase the sugar content to meet the demands of both cell growth and malic acid production. However, the inhibitor content will be enhanced along with the increased sugar contents of the hydrolysates. As shown in Table 1, the corncob hydrolysate was concentrated to a total sugar concentration of 150 g/L, containing approximately 96 g/L glucose, 54 g/L xylose, 0.14 g/L furfural, 2.34 g/L HMF, 0.1 g/L formic acid and 1.8 g/L acetic acid. Compared with the other pretreatment methods [22], the content of HMF in corbcob hydrolysates was relatively low. It is noted that evaporation also removed significant amounts of volatile compounds such as acetic acid and furfural generated from the dehydration of xylose (data no shown). The high glucose and xylose content with little acids makes corncob hydrolyaste a suitable carbon source for malic acid fermentation.

To investigate the effect of hydrolysate sugar concentration on cell growth and malic acid production, initial sugar concentrations from 50 to 130 g/L were tested as shown in Table 2. Increased residual sugar concentration was beneficial for cell growth within the range of 50 to 110 g/L, but malic acid fermentation was inhibited when the concentrated hydrolysate for an equivalent sugars exceeded 110 g/L. Increasing the sugar concentration further did not seem to increase malic acid production because the fermentation was probably inhibited by the accumulation of inhibitors in the condensed hydrolysates. Based on the sugar consumption, the highest malic acid production of 15.27 ± 2.18 g/L was obtained when the initial sugar concentration was 110 g/L.

### Effect of nitrogen sources on cell growth and malic acid production

Six organic and inorganic nitrogen sources including yeast extract, peptone, NH_4_NO_3_, NH_4_H_2_PO_4_, (NH_4_)_2_SO_4_, and NaNO_3_ were evaluated for their effects on the production of malic acid. The results (Table 3) suggested that (NH_4_)_2_SO_4_ was better than the other nitrogen sources for the production of malic acid. Without adding any nitrogen as the control, the concentration of malic acid produced by the natural hydrolysate of corncob fermentation was 18.41±0.29 g/L. The maximum production of malic acid reached 36.24±0.65 g/L when (NH_4_)_2_SO_4_ was used as the nitrogen source. Therefore, (NH_4_)_2_SO_4_ was selected as the nitrogen source for the remaining research.

**Table 3:** Effects of nitrogen source on malic acid production under the hydrolysate of corn cob 110 g/L as the carbon source in shake flasks.

Nitrogen source	Residual sugar (g/L)	Cell biomass (g/L)	Malic acid(g/L)	Productivity(g/L h)	Yield(g/g)
NH_4_NO_3_	12.38	24.82±0.52	30.01±2.52	0.25±0.021	0.31
NaNO_3_	10.56	25.36±0.96	27.50±2.08	0.23±0.017	0.28
(NH_4_)_2_SO_4_	9.98	25.48±1.22	36.24±0.65	0.30±0.005	0.36
NH_4_H_2_PO_4_	11.58	27.03±2.28	24.55±0.29	0.20±0.001	0.25
Yeast extract	13.20	26.44±0.98	23.83±2.61	0.19±0.016	0.25
Peptone	12.76	26.84±1.46	22.24±1.47	0.17±0.023	0.23
Control	11.12	28.12±3.02	18.41±0.29	0.15±0.002	0.19

### Repeated-batch immobilized fermentation for culture adaption

**Fig 1:**
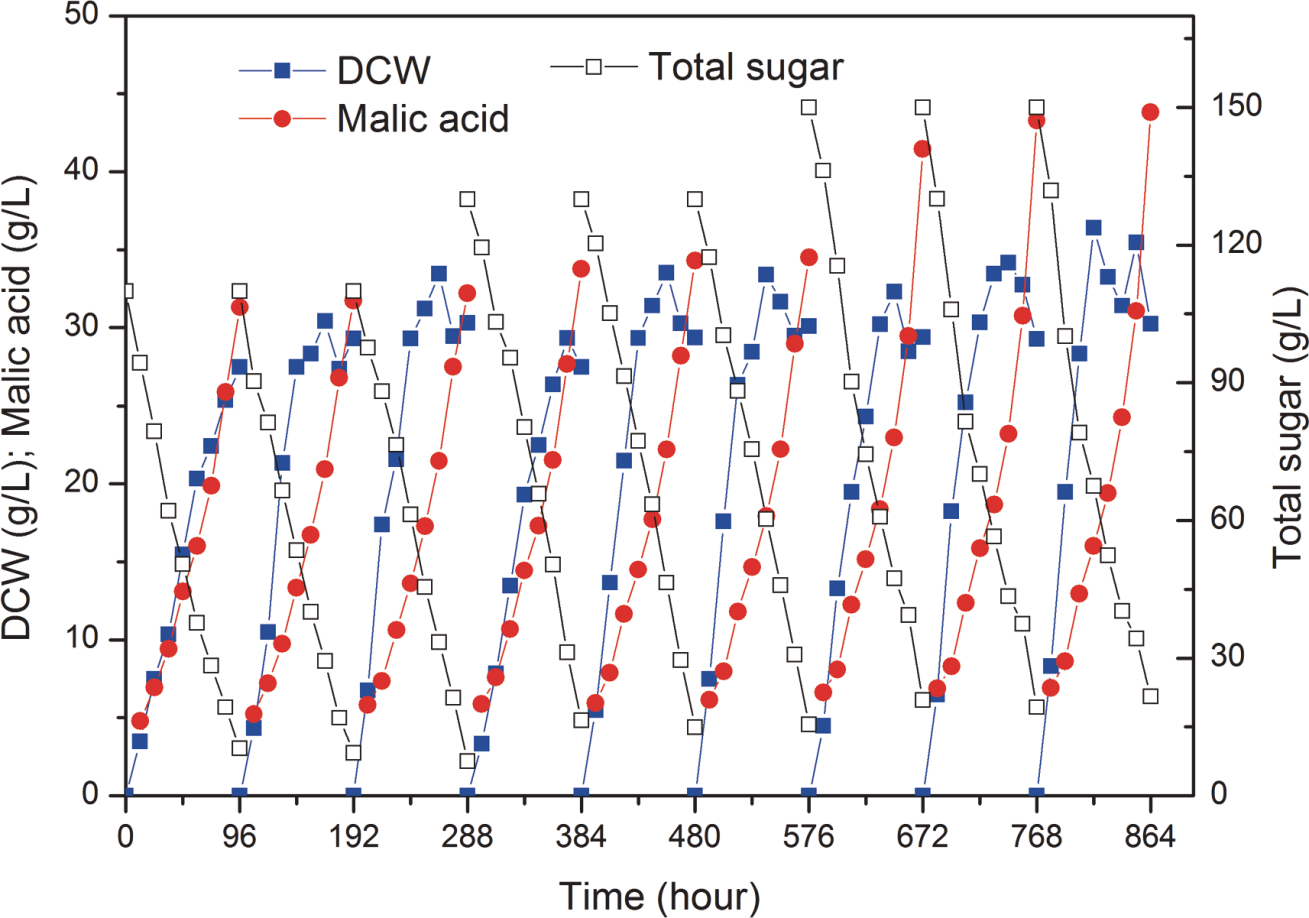
Repeated-batch immobilized fermentation for culture adaption in the AFBB. Date are given as triplicate.

**Fig 2:**
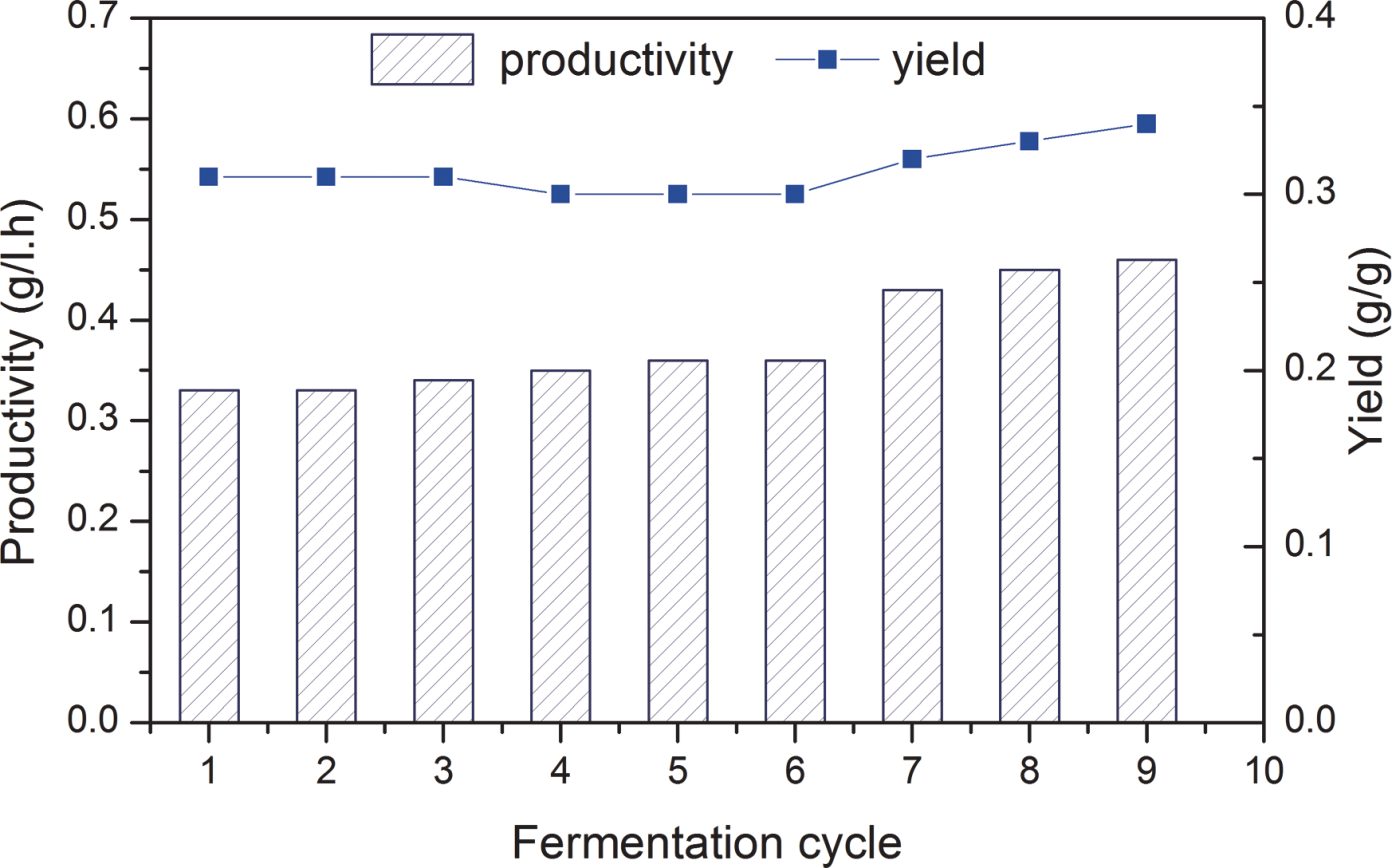
The profiles of malic acid productivity and yield in fermentation cycle. Date are given as triplicate.

The purpose of the experiment was to allow cells to gradually adapt to the high-inhibitor microenvironment in order to evaluate the maximal malic acid concentration that can be produced in fermentation. As seen in Fig. 1, the immobilized cell fermentation in the AFBB produced more malic acid through gradually enhancing the hydrolysate sugar concentration from 110 to 150 g/L. At the end of the ninth batch, the malic acid concentration reached 38.6 g/L, which was higher than that in the first batch (27.6 g/L). The higher malic acid concentration obtained in the batches of immobilized fermentations indicated that the adapted cells in the AFBB were possibly less sensitive to inhibitors stress. The productivity and yield of malic acid in the ninth batch of fermentation were 0.4 g/L h and 0.3 g/g, respectively, which were higher than that in the first fermentation batch (0.29 g/L h and 0.28 g/g, respectively) (Fig. 2). These results clearly indicated that the adapted cells immobilized in the AFBB acquired an ability to tolerate and produce high malic acid concentrations. Therefore, additional experiments were conducted to study the underlying changes or causes. To determine if there were any phenotypic changes in the long-term adapted culture, cells in the AFBB were removed and grown on gradient plates to test their inhibitor tolerance.

### Effect of AFBB adaptation on inhibitor tolerance

**Fig 3:**
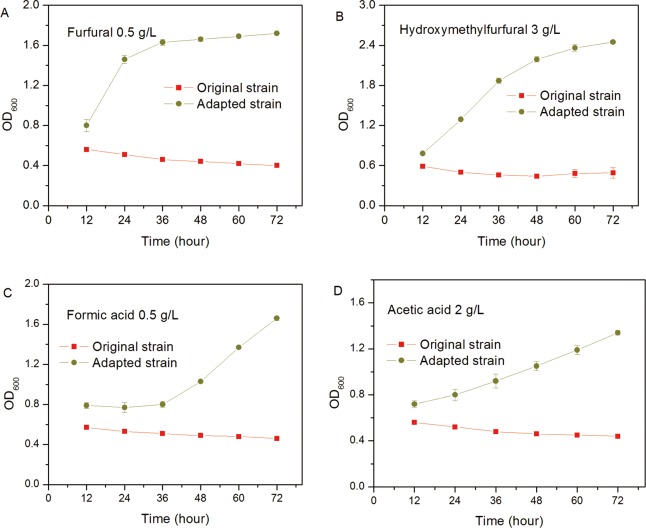
Inhibition effects of different inhibitors on cell growth of the original and adapted strain. Date are given as triplicate. (A) Furfural 0.5 g/L. (B) HMF 3 g/L. (C) Formic acid 0.5 g/L. (D) Acetic acid 2 g/L.

To determine the inhibitor tolerance in both the original and adapted strains, cells were grown as free cell suspension cultures at the minimal inhibitory concentration of different inhibitors. As shown in Fig. 3, after AFBB adaptation of the culture, cell tolerance to furfural, HMF, acetic acid, and formic acid was significantly increased in the adapted strain. Upon feeding 0.5 g/L of furfural, 3 g/L of HMF, 2 g/L acetic acid, and 0.5 g/L of formic acid into the growth medium, the adapted type retained high growth rate, whereas the original strain lost its ability to grow.

**Fig 4:**
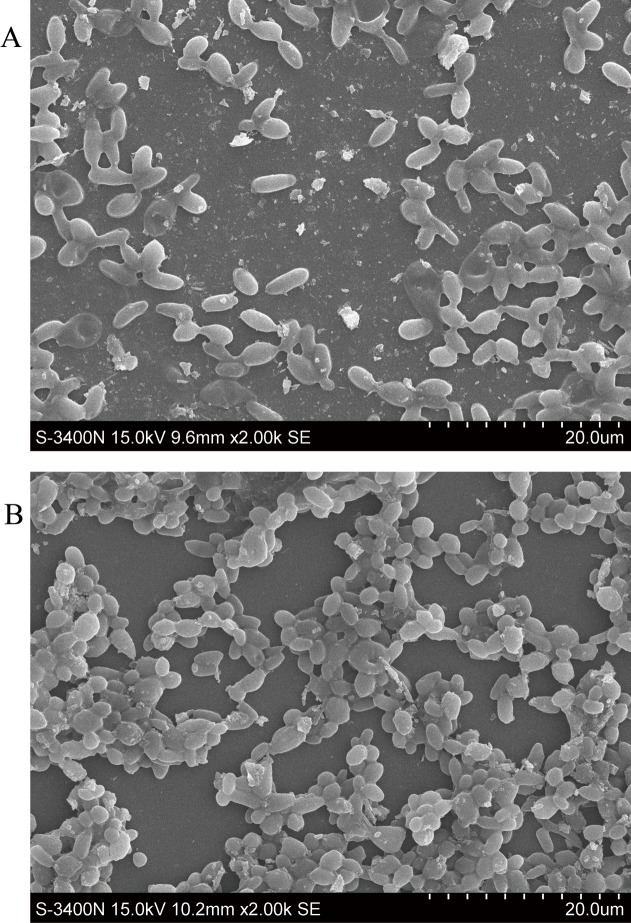
Scanning electron microscope of *A*. *pullulans* showing morphological difference between the original and adapted strain in the stationary phase. Date are given as triplicate. (A) Original cells with a long rod shape. (B) Adapted strain with a short round shape.

In general, the adaptation of microorganisms in an FBB induces some changes in both biochemical characteristics and cell morphology. It has been reported that *Clostridium tyrobutyricum* lost its original rod shape and became longer under repeated fed-batch fermentation of glucose in an FBB [23]. Zhang et al also showed that a propionic acid-producing *Propionibacterium acidipropionici* strain elongated its rod morphology after about 3 months adaptation in an FBB [24]. To investigate the effects of AFBB adaptation on cell morphology of *A*. *pullulans*, adapted and original cells in the stationary phase were harvested and observed with scanning electron microscopy. As can be seen in Fig. 4, the original strain had a rod shape, but the mutant appeared to be much shorter and thinner than the wild type, and part of the mutant gradually became a spherical shape as observed with scanning electron microscopy. The adapted strain had an average length of 2 μm (vs. 4 μm for the original type). Compared to the wild type, the approximately spherical appearance of the adapted strain had an obvious increase in its specific surface area and cell density per unit area in the AFBB, which might contribute to the proportional increases in substrate uptake efficiency and metabolite excretion rates.

### Transcriptome response to furfural and HMF stress

**Fig 5:**
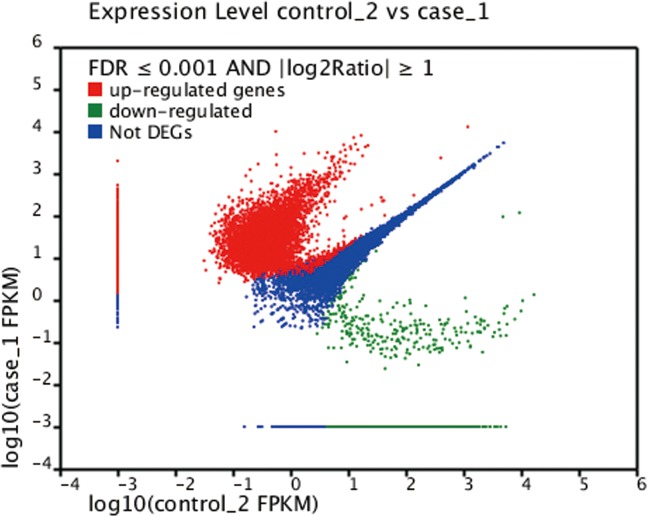
Volcano plot result from genomic analysis showing significantly differentially expressed genes under furfural and HMF stress condition. *Green dots* indicate down-regulated genes and *red dots* indicate up-regulated genes. *Blue-colored* were not considered as significantly differently expressed. The *X*-axis shows the difference values of the control group based on a log_10_ scale. The *Y*-axis shows statistical significance values of the furfural and HMF treated group based on a log_10_ scale.

We investigated the cellular response to the presence of furfural and HMF on a global transcriptional level. Based on the genome data of *A*. *pullulans*, the global transcriptional response of the original strains and strains adapted to furfural and HMF stress were examined at 12 h after inoculation to induce stress conditions (media with 0.5 g/L furfural and 3 g/L HMF) and compared with unstressed, normal conditions (media without furfural and HMF). In this time nodes, cell growth was in the lag phase as shown in Fig. 3, and could be obviously affected by the stress eniviroment. In the original strain, we obtained 66,136 contigs with length ≥200 bp. The N50 length of these contigs was 1,024 bp, and the N90 number was 148 bp. There were 8,605 contigs with length ≥1,000 bp and 2,614 contigs with length≥2,000 bp. In addition, 45,462 contigs with length ≥200 bp was identified in adapted strain. The N50 length of these contigs was 1,300 bp, and the N90 number was 206 bp. There were 8,206 contigs with length ≥1,000 bp and 2,795 contigs with length≥ 2,000 bp. Identified as being significantly upregulated were 14,809 unigenes and 21,745 unigenes were downregulated during furfural and HMF stress condition (Fig. 5).

**Fig 6:**
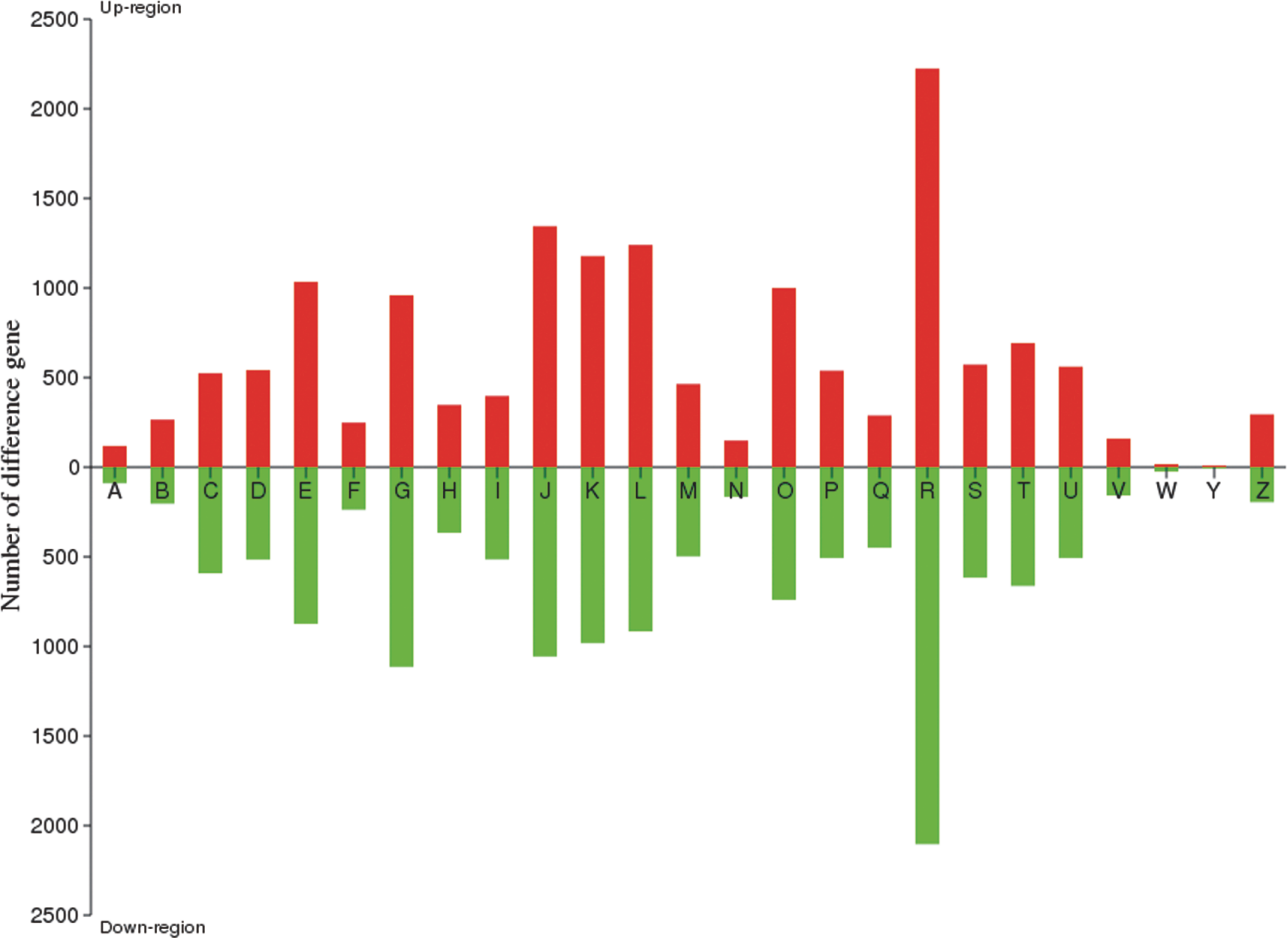
Number of differentially expressed genes according to the *A*. *pullulans* genome database. Columns: *A* RNA processing and modification; *B* chromatin structure and dynamics; *C* energy production and conversion; *D* cell cycle control, cell division, chromosome partitioning; *E* Amino acid transport and metabolism; *F* Nucleotide transport and metabolism; *G* Carbohydrate transport and metabolism; *H* Coenzyme transport and metabolism; *I* Lipid transport and metabolism; *J* Translation, ribosomal structure and biogenesis; *K* Transcription; *L* Repication, recombination and repair; *M* Cell wall/membrane/envelope biogenesis; *N* Cell motility; *O* Posttranslation modification, protein turnover, chaperones; *P* Inorganic ion transport and metabolism; *Q* Secondary metabolites biosynthesis, transport and catabolism; *R* General function prediction only; *S* Function unknown; *T* Signal transduction mechanisms; *U* Intracellular trafficking, secretion, and vesicular transport; *V* Defense mechanisms; *W* Extracellular structures; *Y* Nuclear structure; *Z* Cytoskeleton.

Fig. 6 provides a summary of the percentage of differentially expressed genes grouped by functional categories according to TIGR’s annotation of the *A*. *pullulans* genome. In the presence of furfural and HMF, about 47.22% of the genes related to carbohydrate transport and metabolism, amino acid transport and metabolism, protein biosynthesis, lipid transport and metabolism, signal transduction mechanism, and energy production and conversion showed greater expression than under normal conditions. Gene ontology (GO) analysis enriched proteins mainly involved in the response to ATP binding (GO: 0005524), cofactor binding (GO: 0048037), oxidoreductase activity (GO: 0016491), carboxylic acid biosynthesis process (GO: 0046394), and organic acid biosynthesis process (GO: 0016053), etc. (Table 4).

**Table 4:** Enriched regulated Gene Ontology Groups in the adapted strain under the furfural and HMF stress.

GOTERM Category	GO number	Term	Count	%	P-value
Biological Process	GO:0042180	Cellular ketone metabolic process	41	5.80	0.000
	GO:0006082	Organic acid metabolic process	19	8.80	0.000
	GO:0019752	Carboxylic acid metabolic process	38	8.80	0.000
	GO:0043436	Oxoacid metabolic process	23	8.80	0.000
	GO:0044283	Small molecule biosynthetic process	15	4.90	0.000
	GO:0044281	Small molecule metabolic process	22	17.90	0.000
	GO:0016053	Organic acid biosynthesis process	16	4.50	0.000
	GO:0046394	Carboxylic acid biosynthesis process	39	4.50	0.000
	GO:0055114	Oxidation reduction process	36	12.00	0.000
	GO:0006520	Cellular amino acid metabolic process	29	6.70	0.000
	GO:0032787	Monocarboxylic acid metabolic process	85	1.80	0.012
	GO:0006631	Fatty acid metabolic process	32	1.30	0.020
Cellular Component	GO:0016020	membrane	14	2.90	0.000
	GO:0005886	Plasma membrane	14	6.90	0.000
	GO:0071944	Cell periphery	6	9.50	0.000
	GO:0016021	Integral to membrane	14	22.20	0.000
	GO:0031224	Intrinsic to membrane	10	22.60	0.001
	GO:0044425	Membrane part	108	27.50	0.001
Molecular Function	GO:0043167	Ion binding	4	41.7	0.000
	GO:0043169	Cation binding	27	17.2	0.000
	GO:0046872	Metal ion binding	10	16.50	0.000
	GO:0030554	Adenyl nuteotide binding	11	15.50	0.000
	GO:0032559	Adenyl ribonucleotide binding	17	15.50	0.000
	GO:0005524	ATP binding	20	15.50	0.000
	GO:0017076	Purine ribonucleotide binding	11	17.90	0.000
	GO:0032553	Ribonucleotide binding	11	17.90	0.000
	GO:0017076	Purine ribonucleotide binding	11	17.90	0.000
	GO:0035639	Purine ribonucleoside triphosphate binding	11	17.80	0.000
	GO:0036094	Small molecule binding	9	28.80	0.000
	GO:0046914	Transition metal ion binding	15	9.00	0.000
		Nucleotide binding			
	GO:0016491	Oxidoreductase activity	87	12.90	0.000
	GO:0048037	Cofactor binding	8	5.30	0.000
	GO:0016747	Transferase activity, transferring acyl groups other than amino-acyl groups	89	1.70	0.001
	GO:0008270	Zinc ion binding	6	2.50	0.010
	GO:0016746	Transferase activity, transferring acyl groups	6	2.50	0.010
	GO:0022892	Substrate specific transport activity	11	7.20	0.020
	GO:0016407	Acetyltransferase activity	14	0.80	0.029
	GO:0005215	Transporter activity	15	9.60	0.033
	GO:0016879	Ligase activity, forming carbon-nitrogen bonds	28	2.40	0.040

After challenging with furfural and HMF, some gene expressions increased by up to 15-fold compared with gene expression in the control group (Table 5). Five genes (Unigene 13926, Unigene 17255, Unigene 22366, Unigene 14386, and Unigene 15065) that were involved in oxidative phosphorylation and four genes (Unigene 14058, Unigene 14702, Unigene 14018, Unigene 21154) that were involved in the TCA cycle were associated with energy and cofactor metabolism. In *S*. *cerevisiae*, the redox metabolism was severely affected by furfural and HMF [25]. In addition, malic acid is an intermediate in the TCA cycle, and upregulation of genes involved in the TCA cycle maybe increase the metabolic flux for malic acid production.

**Table 5:** Genes up-regulated by more than 15 folds in the adapted strain under the furfural and HMF stress.

Unigene number	Gene Name	Fold Change	Pathway
13926	NADH dehydrogenase	17.5	Oxidative phosphorylation
17255	Ubiquinol-cytochrome c reductase cytochrome b/c1 subunit	17.1	Oxidative phosphorylation
22366	F-type H+-transporting ATPase subunit	17.1	Oxidative phosphorylation
14386	Cytochrome c oxidase cbb3-type subunit I	16.2	Oxidative phosphorylation
15065	NADH-ubiquinone oxidoreductase chain 4L	15.8	Oxidative phosphorylation
14058	Pyruvate dehydrogenase E2 component	17.6	TCA cycle
14702	Isocitrate dehydrogenase	16.6	TCA cycle
14018	2-oxoglutarate dehydrogenase E2 component	15.5	TCA cycle
21154	Fumarate hydratase	15.3	TCA cycle
22163	Methylsterol monooxygenase	18.6	Steroid biosynthesis
18887	Cytochrome P450, family 51 (sterol 14-demethylase)	17.1	Steroid biosynthesis
19394	Sterol 24-C-methyltransferase	15.7	Steroid biosynthesis
22559	3-oxoacyl-[acyl-carrier protein] reductase	15.7	Fatty acid biosynthesis
19017	Acetyl-CoA carboxylase	15.2	Fatty acid biosynthesis
12121	Calmodulin	18.5	Phosphatidylinositol signaling system
21176	Inositol-1,4,5-trisphosphate 5-phosphatase	15.0	Phosphatidylinositol signaling system
19354	Dihydroxyacetone kinase	16.3	Glycerolipid metabolism
10417	Triacylglycerol lipase	16.0	Glycerolipid metabolism
14508	1-acyl-sn-glycerol-3-phosphate acyltransferase	15.9	Glycerolipid metabolism
18484	Trehalose 6-phosphate synthase	15.9	Starch and sucrose metabolism
10819	Glycogen debranching enzyme	15.0	Starch and sucrose metabolism
22225	Phosphoglucomutase	16.8	Starch and sucrose metabolism
21978	P21-activated kinase 1	15.0	MAPK signaling pathway
10439	Osomolarity two-component system, response regulator SSK1	15.2	MAPK signaling pathway
21610	Zinc finger protein MSN2/4	15.1	MAPK signaling pathway
21803	ATP-binding cassette, subfamily G (WHITE), member 2, PDR	16.0	ABC transporters
10754	Transcription initiation factor TFIIE subunit beta	15.1	Basal transcription factors
22534	Small subunit ribosomal protein S20e	16.1	Ribosome
22171	Large subunit ribosomal protein L4e	16.1	Ribosome
22657	Small subunit ribosomal protein S3e	15.5	Ribosome
12327	Large subunit ribosomal protein L26e	15.9	Ribosome
12393	Large subunit ribosomal protein L11e	17.0	Ribosome
22501	Small subunit ribosomal protein S14e	16.4	Ribosome
22571	Large subunit ribosomal protein L30e	15.8	Ribosome
22393	Large subunit ribosomal protein L40e	16.3	Ribosome
22124	Small subunit ribosomal protein S27e	18.0	Ribosome
14476	Gamma-glutamyltranspeptidase	17.0	Glutathione metabolism
14702	Isocitrate dehydrogenase	16.6	Glutathione metabolism
19360	Glutathione reductase	15.9	Glutathione metabolism
22582	Spermidine synthase	15.7	Glutathione metabolism
10353	Cystathionine gamma-lyase	16.5	Cysteine and methionine metabolism
19280	Kynurenine-oxoglutarate transaminase	15.0	Tryptophan metabolism

It has been reported that microorganisms can increase their tolerance to organic solvents by regulating their membrane lipid composition in response to environmental stresses [23,26]. Yang et al. found that the increase of fatty-acyl-chain length of phosphatidylcholine and phosphatidylinositol in *S*. *cerevisiae* had a strong correlation with high furfural, acetic acid, and phenol tolerance seen with phospholipidomics and transcriptomics analyses [27]. In the adapted *A*. *pullulans* strain, eight genes (Unigene 22163, Unigene 18887, Unigene 19394, Unigene 22559, Unigene 19017, Unigene 19354, Unigene 10417, and Unigene 14508) involved in steroid biosynthesis, fatty acid biosynthesis, and glycerolipid metabolism were related to lipid metabolism (Table 4). Moreover, fatty acid composition in cell membrane would change the membrane fluidity and probably also regulate the membrane permeability or cell morphology [23]. This indicated that the changes of cell morphology in adapted strain maybe attribute to the difference of lipid metabolism.

**Fig 7:**
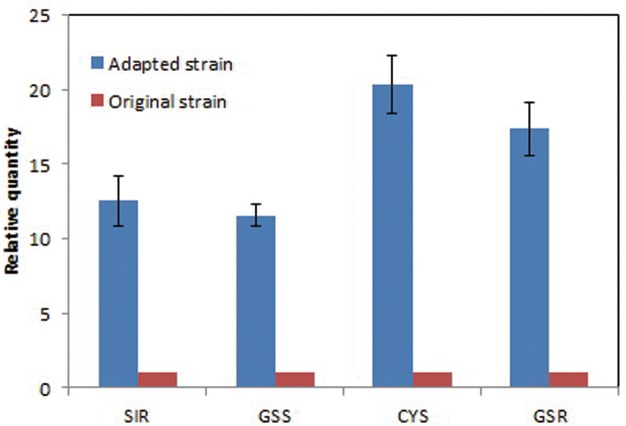
Transcription levels of sulfite reductase(SIR), glutathione synthase (GSS), cysteine synthase (CYS), and glutathione reductase (GSR) involved in sulfur assimilation pathway between the original and adapted strain. Date are given as triplicate.

Nine ribosomal protein genes (Unigene 22534, Unigene 22171, Unigene 22657, Uunigene 12327, Unigene 12393, Unigene 22501, Unigene 22571, Unigene 22393, and Unigene 22124) were associated with protein biosynthesis. Five genes (Unigene 14476, Unigene 14702, Unigene 19360, Unigene 22582, and Unigene 10353) involved in glutathione (GSH) and cysteine metabolism were associated with redox metabolism. The redox status of protein sulfhydryl groups is mainly regulated by glutaredoxins (GRXs) and thioredoxins (TRXs), which are reduced by GSH- and NADPH- dependent thioredoxin reductase, respectively [28]. The expression of Unigene 14702 encoding isocitrate dehydrogenase increased by up to 16.6-fold, indicating the adapted strain produced more cofactor NADPH for responding to furfural and HMF stress. In *E*. *coli*, NADPH-dependent reductases are widely used for converting furfural to the less inhibitory alcohol [10,29]. Furthermore, furfural and HMF, acting as thiol-reactive electrophiles, have been shown to induce the accumulation of reactive oxygen species (ROS), leading to damage of cell components, including mitochondrial and vacuolar membranes and chromatin in yeast [30]. Recently, Kim et al. found that increasing GSH levels by overexpression of genes for glutathione biosynthesis (*GSH1* and *GLR1*) enhanced tolerance to furfural in *S*. *cerevisiae* [31]. Therefore, the expression levels of the key genes involved in sulfur assimilation pathway were further confirmed by qRT-PCR as shown in Fig. 7. The expression level of four genes i.e. sulfite reductase (SIR), glutathione synthase (GSS), cysteine synthase (CYS), and glutathione reductase (GSR) in adapted strain were unregulated by over 10-fold compared to that of the original strain. These results indicated that the sulfur assimilation pathway in adapted strain was activated for cellular protection against oxidative stress.

It is interesting that five genes (Unigene 12121, Unigene 21176, Unigene 21978, Unigene 10439, and Unigene 21610) involved in the phosphatidylinositol signaling system and mitogen-activated protein (MAPK) signaling pathway were correlated with signal transduction. In eukaryotic cells, MAPK pathways are of central importance because they are critically involved in controlling cell growth and differentiation as well as in establishing stress response [32]. The results indicated that an engineered MAPK pathway might be an effective method of increasing inhibitor tolerance.

## Conclusion

Corncob is abundant agricultural residues in China, although some corncobs are used to produced xylitol, a large amount of corncobs or corncob cellulosic residues are regarded as solid wastes [33]. In this study, the process for producing malic acid from a hydrolysate of corncob was investigated with a polymalic acid (PMA)-producing *A*. *pullulans* strain. Under the initial hydrolysate sugar concentration 110 g/L, and (NH_4_)_2_SO_4_ 2 g/L, malic acid production in a shake flask was improved. The maximum production of malic acid reached 36.24±0.65 g/L, which was 49.2% higher than that of natural hydrolysate of corncob fermentation. The results revealed that *A*. *pullulan* has a good ability to utilize the biomass feedstocks.

In our previous work, based on the aerobic characteristic of the *A*. *pullulans* culture, we constructed an aerobic fibrous-bed bioreactor (AFBB) for malic acid fermentation [20]. Using the adapted evolution strategy in an AFBB, The productivity and yield of malic acid in the ninth batch of fermentation were 0.4 g/L h and 0.3 g/g, respectively, which were higher than that in the first fermentation batch (0.29 g/L h and 0.28 g/g, respectively). Moreover, after AFBB adaptation of the culture, *A*. *pullulans* tolerance to furfural, HMF, acetic acid, and formic acid was significantly increased. Upon feeding 0.5 g/L of furfural, 3 g/L of HMF, 2g/L acetic acid, and 0.5 g/L of formic acid into the growth medium, the adapted mutant retained high growth rate, whereas the original strain lost its ability to grow. The results indicated that *A*. *pullulans* mutant strain improved its level of malic acid production, and enhanced inhibitor tolerance present in corncob hydrolysate.

Transcriptomic studies performed in this study provided a new multifarious basis for the inhibitory effects of furfural and HMF on *A*. *pullulans*. The differentially expressed genes were related to carbohydrate transport and metabolism, protein biosynthesis, lipid transport and metabolism, signal transduction mechanism, redox metabolism, and energy production and conversion. qRT-PCR further confirmed that the sulfur assimilation pathway in adated strain was activated for cellular protection against oxidative stress. To summarize, this study provides insights that can form the basis for metabolic engineering of *A*. *pullulans* for improving bioconversion of lignocellulose biomass hydrolysates into malic acid production.

## Supporting Information

S1 Table Primers for the gene transcription level analysis. (DOC)
