# Supplementary material for: Adaptation and Transcriptome Analysis of Aureobasidium pullulans in Corncob Hydrolysate for Increased Inhibitor Tolerance to Malic Acid Production
Source: PLoS One. 2015 Mar 20;10(3):e0121416. doi: 10.1371/journal.pone.0121416 (PMC4368199; doi:10.1371/journal.pone.0121416)
Supplement: S1 Table — (DOC) [file pone.0121416.s001.doc]

**S1 Table** Primers for the gene transcription level analysis

| Genes | Primer names | Primer sequence | Size  (bp) |
| --- | --- | --- | --- |
| Sulfite reductase | SIR-F | CAAGCACAGCAGCCAACA | 91 |
|  | SIR-R | CGATCCGCATATCCAGAAT |  |
| Glutathione synthase | GSS-F | CTTCCAGACTTGGTTGATGC | 220 |
|  | GSS-R | CACCCCATTCGCTTTCAT |  |
| Cysteine synthase | CYS-F | CATCGTCACCAACTCCTCG | 201 |
|  | CYS-R | CAAGATTGTTTGTGCGGGTA |  |
| Glutathione reductase | GSR-F | GCTTCGTTGGTGGTTTCG | 175 |
|  | GSR-R | AGCCGTTTCTGGTAGTGG |  |
| 18S rRNA | 18S-F | GTTGGTGGAGTGATTTGTCTGC | 252 |
|  | 18S-R | GCACGACGGAGTTTCACAAGAT |  |
